# The Earliest Report

**Published:** 1924-04

**Authors:** 


### "The Earliest Report."

Sir,—With reference to the paragraph under this heading in your March issue (page 84), may I be allowed to ask of the Manchester Royal Infirmary the following questions:—

1. Are the accounts kept upon the income and expenditure system of accounting? 2. Are stores accounts kept, or are purchases treated as expenditure and analysed direct from the cash payment? 3. Are liabilities incurred, but not paid, recorded in the books? 4. Are liabilities outstanding at the end of the year brought into account, and included in the balance-sheet? 5. Are items of income recorded in the books before receipt? 6. Are amounts outstanding due from debtors at the end of the year brought into account, and included in the balance-sheet as assets? 7. Are separate records kept of the expenditure on in-patients and out-patients respectively? 8. If separate records are not kept, upon what basis are the in-patient and out-patient statistics prepared?

Inquirer.

